# Active Ageing Level and Time Use of Elderly Persons in a Thai Suburban Community

**DOI:** 10.1155/2019/7092695

**Published:** 2019-01-22

**Authors:** Autchariya Punyakaew, Suchitporn Lersilp, Supawadee Putthinoi

**Affiliations:** Department of Occupational Therapy, Faculty of Associated Medical Sciences, Chiang Mai University, 110, Intawaroroj Rd., Sripoom Subdistrict, Meung, Chiang Mai 50200, Thailand

## Abstract

Elderly populations are growing rapidly worldwide, thus enhancing an increasing need for their independent health care, productivity, and most importantly, balance of occupations. This research is aimed at investigating the active ageing levels and time use patterns of an elderly population attending the Community Elderly School in a suburban village of northern Thailand. These participants comprised 140 persons aged 60 years and older and were without cognitive deficits, based on the Mini-Mental State Examination. Active ageing levels and time use patterns were collected by following an interview-based questionnaire. The results showed that the level of active ageing was moderate (mean active ageing index (AAI) was 0.79). All of the participants used their time in varied activities, including the seven categories: basic activities of daily living, instrumental activities of daily living, rest and sleep, education, work, leisure, and social participation. Furthermore, they spent most of their time resting and sleeping.

## 1. Introduction

The proportion and number of elderly people are increasing dramatically worldwide, and the tendency for people to live longer is associated with economic and social development [1]. When focusing on the ageing situation in Asia, Thailand is ranked as having the second most rapidly growing ageing population, being almost faster than that in developed countries [2]. In 2005, Thailand entered the ageing society, meaning that the percentage of elderly aged 60 and over had reached 10% of the country's overall population. It is expected that Thailand will be faced with an aged society in 2021 [3]. This means that Thailand has a very short time to prepare a response to demographic change. Therefore, economic and social restructuring, including collaboration of all sectors, will need to support the elderly society and respond to this situation.

The World Health Organization (WHO) established the active ageing concept in 2002 [4] in order to respond to the rapidly growing number of persons aged 60 and over. Its aim is at preserving the active role of elderly persons in society, with continued opportunity for health care, social participation, and security. Numerous changes occur with increasing age such as the risk of chronic disease [5, 6]; therefore, promoting, preparing, and planning for future ageing may be accompanied by improved quality of life, well-being, and independence, including elderly people being able to maintain autonomy.

Research on active ageing in Thailand has been scarce. Previous studies focused on investigating the active ageing level and factors predicting active ageing in order to understand personal factors, knowledge about ageing, attitude toward ageing, and social support. The active ageing level of Thai elderly was classified as moderate in 2002 [7], 2014 [8], 2015 [9], and 2016 [3]. A study by Kespichayawattana and Wiwatvanich [10] explored the attributes of active ageing in healthy and well-known Thai elderly by using a case study with a qualitative approach. Three attributes consisted of being active continually, being healthy, and having security.

A study of determinants for active ageing in the Thai context was conducted based on elements of health, community participation, and security. It was found that the Thai active ageing levels were on age, area of residence, education, marital status, social support, gender, income, work, health checkup, ministered care, and exercise [11, 12]. The concept of active ageing is complex in not only health indicators but also social, environmental, and economic aspects, which vary between contextual and cultural perspectives, and this should be considered [6]. Thanakwang et al. [13] focused on understanding active ageing in the Thai cultural context, with some dimensions of it being distinct from those in a Western one such as specifically spiritual growth (intrapersonal strength and calmness) and managing security in later life (being cared for by strengthening family ties). Thus, the study of active ageing should contribute to implementing proper interventions at the policy level.

Adoption of the WHO concept of active ageing in Thailand should be applied and implemented in the Thai context. Each dimension of active ageing (health, community participation, and security) is in keeping with a healthy lifestyle and a structure that uses a weighted score for an active ageing indicator [7]. However, Danyuthasilpe et al. [14] revealed that the way of leading a healthy ageing lifestyle is to eat heathy food, relate with cultural roots, and practice physical activity (exercise). Studies that focused on positive ageing such as healthy, successful, and productive ageing in Thai elderly also were limited.

Promoting healthy ageing, in lifestyle or social support for instance, is a good implementation strategy for reducing the burden of age-related disease [15]. In order to have a healthy lifestyle, varied activity in daily routine is important. Time use through day-to-day activities is a link to health and well-being. There have been very few studies on active ageing and time use in Thailand. Therefore, the study of time spent by elderly people in routine and active ageing is needed and would contribute to improving their quality of life. Daily activities for elderly persons are important in providing full occupation and bringing a meaning and purpose to life [16]. The elderly have engaged in varied activities of daily life since their human occupation involvement was linked inextricably with time. Therefore, this study is aimed at exploring the active ageing levels and time use in healthy elderly people, which embraces a positive outcome with the challenge of fostering their well-being and quality of life.

## 2. Materials and Methods

### 2.1. Study Design

This study was a cross-sectional survey of elderly people that provided data on the active ageing level and time use patterns in a study area. A general image of time used by the elderly in an ordinary day was assessed in the research.

### 2.2. Ethical Approval

This study was approved by the Ethics Committee of the Faculty of Associate Medical Sciences, Chiang Mai University (number: AMSEC-61EX-007).

### 2.3. Study Setting and Participants

The study setting was at Sannameng village, Chiang Mai, northern Thailand. The community there was selected because it is in an area under cooperative activity in clinical placement of the Occupational Therapy Department, Chiang Mai University. This study consisted of 140 elderly participants from a list of student members in the Community Elderly School. The inclusion criteria were being aged 60 years and older, being voluntary participants in the study, and being cooperative in their ability to understand the questionnaire. The exclusion criterion was cognitive deficit found by using the Mini-Mental State Examination (scored according to the education of the participant) [17]. All of the participants gave their informed consent to take part in this study.

### 2.4. Data Collection and Analysis

The data were collected from 1 December 2017 to 30 April 2018. Researchers visited the participants at the Community Elderly School to explain the purpose of the study and collect basic data, including sociodemographics and cognitive function. The active ageing index (AAI) was calculated using a weighted score for each active ageing dimension (health, community participation, and security) [7]. Next, each participant was selected on a day for collecting data about their time use by using the recall time diary method [18]. Each individual participant met the researcher twice. Time use data were collected at the first meeting through a semistructured interview to recall the sequence of activities. The systematic recall, or people to recall activities measure, of how the elderly spend their days in each individual activity from the beginning to end of a 24-hour period was used. At the second meeting, time use data concerned with what influence disturbance had on atypical days were collected 2 times per person by following the study of Krupa et al. [19].

### 2.5. Data Analysis

Raw scores were calculated to obtain AAI scores from all of the participants by following the formula: active ageing index = 1/3 (health index) + 1/3 (participation index) + 1/3 (security index) [7], where scores ranging from 0 to 1 and a higher value of AAI indicated a higher active ageing level (>0.5 = low level, 0.5>−0.79 = moderate level, and >0.8 = high level) [20]. The whole series of personal activities during the 24-hour period was considered as the units of analysis: number of minutes to the number of hours from the start time to the end. The raw data of activities were grouped into items of eight major categories: activities of daily living (ADL), instrumental activities of daily living (IADL), rest and sleep, education, work, play, leisure, and social participation. These categorizations were based on the occupational therapy practice framework: domain and process [21]. Next, comparison of daily time use and activity between this study of Thai elderly people and other studies of different ageing populations was analyzed. Items of activities were grouped based on the occupational therapy domain and process for analytic purposes. Time spent in each category was calculated by the hour in order to be possible theoretically for a given data. Then, each categorization was investigated for performance while being alone or with others, indoors or outdoors. Several time uses constructed a specific level of active ageing in the healthy Thai elderly. For a better understanding, each active ageing group level (high, medium, and low) was identified for the time spent (over 24 hours) in each category of activity.

## 3. Results

Elderly people from the Community Elderly School were investigated in Sannameng Subdistrict. A total of 145 elderly people agreed to participate in this study. However, 5 participants were excluded, due to missing data on their time use. Thus, 140 elderly people (96.55% of the target population) were participants. Demographics and the AAI were analyzed by descriptive statistics to calculate frequency, percentage, and mean and standard deviations. The chi-square test was used to analyze differences in demographic variables and the AAI. The results were divided into three parts: demographic profile of the elderly, AAI, and time use analysis.

### 3.1. Sociodemographic Information

All of the elderly people were Buddhist. The number of females was higher than that of males (79.29% and 44%, respectively). The majority of 79.29% was at least high school graduates. The characteristics of the participants are described in Table 1.

### 3.2. Active Ageing Levels

The attributions of active ageing among elderly Thai persons in the community are shown in Table 2. Fifty-eight of the participants scored a high level of active ageing (AAI scores between 0.80 and 1), with 40% scoring at the moderate level (AAI scores between 0.52 and 0.79), and the remaining 2% at the low level (AAI scores between 0.38 and 0.49). The mean score among this AAI group was at the moderate level.

Active ageing levels were reported among the demographic data (Table 3). The factor that resulted in gender attribution was more likely to be reported differently between elderly females and males. In addition, active ageing levels were not different in other factors: age, marital status, education, health status, employment, and family status.

### 3.3. Time Use

**Figure 1:**
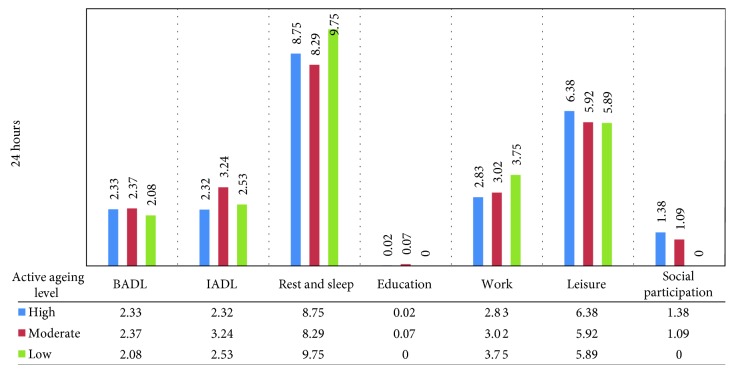
Time spent in each category of activity among three active ageing levels of the elderly.

Daily activities among elderly people in the Community Elderly School were investigated. Activities ranged from 0.25 to 13.50 hours on average per day. The complete results for time spent in each activity are given in Table 4 and Figure 1. Comparison of time use is summarized in Table 5.

**Figure 2:**
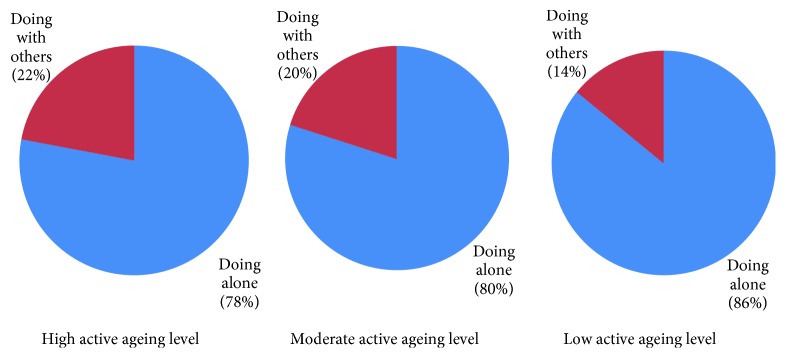
Percentage of activity performance while being alone or with others among three active ageing levels.

**Figure 3:**
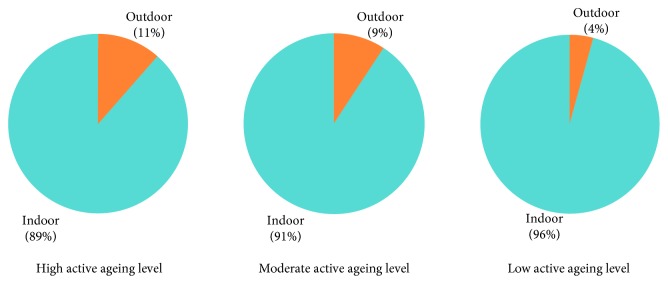
Percentage of activity performance outdoors or indoors among the three active ageing levels.

Regarding total activity performance, which was analyzed according to the three active ageing levels (high, moderate, and low), the participants spent their time alone or with others, as shown in Figure 2. It was found that the elderly engaged in their activity with others at the moderate level, which tended to move toward high. In terms of geographic context, results revealed that a high level (11%) of the participants spent their time outdoors, followed by those at the moderate (9%) and low (4%) levels, as shown in Figure 3.

Most outdoor activities were found to be varied such as shopping, going to the temple, bicycling, attending cremation, and volunteering. All of the elderly people were occupied in outdoor activity, with the average at 10.8% of the total time (24 hours).

## 4. Discussion

It was found that healthy elderly people in the Community Elderly School attributed to active ageing at the moderate level, according to the AAI (mean AAI of the total = 0.79). Despite increasing interest in active ageing in Thailand, the current active ageing level is not as high as that studied previously in 2006 [7]. Nevertheless, the dramatic increase in number of elderly people in Thailand is very challenging. Thus, appropriate strategies are required to deal with this problem. However, when considering percentages in this study, elderly persons tended to represent moderate to high levels. This might reflect on the type of participants in this study, who were students in the Community Elderly School or healthy people. The Thai government has implemented the 2nd National Plan on the Elderly (2002-2021) [24] in order to encourage and develop the education service and lifelong learning, as well as establish and run clubs and networks for the elderly. The Community Elderly School has established a service provided for the elderly under the responsibility of the local government [25]. Thus, the school, as a center of activities, is aimed at providing health promotion activities (local wisdom transfer and cultural or recreational activity) rather than academic action that focuses on socially bound elderly groups. Moreover, this finding has significantly different AAI scores between males and females, which is in accordance with many prior studies [3, 7, 9]. It is true that the active ageing level depends on gender in Thailand.

Time use on specific activities is classified into categories under the framework of domain and process [21], which believes that everyday life activities of people enhance well-being. The elderly people in school in this study spent most of their time resting and sleeping for 8.58 hours a day on average. Similar to several studies in Western society [16, 26], the majority of elderly people spent their daytime sleeping. Sufficient duration of sleep is essential in promoting optimal health in adults, as it involves many functions such as immune function and cardiovascular, metabolic, and mental health [27]. The National Sleep Foundation (NSF) [28] recommends common sleep time for senior adults of about 7 to 9 hours a day. The comparison of time use in each category was difficult in earlier studies because of different categorizing data. Thus, the occupational therapy domain and process [21] provides a description that compares activities in varied detail. The mean time spent in basic ADL and rest and sleep in this study was similar when compared with other countries. It was more difficult to find comparisons in leisure and social participation because these activities (phone and computer use, recreation, community activities, religious activities, and travel) were aggregated. For example, older people in France [22] spent more time working than in other countries because young-old French adults with active life engagement were studied.

However, the elderly with a high active ageing level in this study spent more time in leisure and social participation than those in the moderate- and low-level groups. Furthermore, elderly people at the higher active ageing level spent more of their time with others outdoors than those in lower groups. This finding supports the fact that active ageing relates to engagement in leisure and social participation. Gauthier and Smeeding [29] revealed that the elderly reallocated their time by decreasing that for work and doing more leisure activities. Thus, leisure is a substitute time use for work when people retire from their job. The benefits of time spent in leisure activities can lead to good health and well-being worldwide [30–32]. Therefore, leisure activities associate with a higher quality of life, as key aspects of the active ageing concept. In addition, it was found that elderly people at the high active ageing level not only spent most of their time on leisure activities but also engaged in social participation. Social participation activities include engagement with others such as those in clubs for the elderly, activities in schools for the elderly, religious activities, and cultural events in the community as well as social contribution (village health volunteer and volunteering for public benefits). Similarly, Thanakwang et al. [13] explained that Thai elderly people involved themselves actively in both formal and informal social activities, which related to their community and society at large. The participation of elderly people in social activities facilitates their potential, dignity, and self-actualization positively [33] as an important indicator of active ageing.

## 5. Conclusions

This study examined the active ageing level of healthy elderly people by evaluating the AAI (range from 0 to 1) and investigating their time use in each active ageing level group. The study results showed that active ageing was at the moderate level (mean AAI = 0.79) and indicated its measure as significantly different (*p* < 0.05) between males and females. This research found that elderly people spent most of their time resting and sleeping (8.58 hours per day). Elderly people at the high level of active ageing engaged in leisure and social participation more than those in other groups (moderate and low active ageing levels). This research aimed at drawing attention to the importance of what way time was spent or used by healthy elderly people in the community, as classified specifically in the active ageing level of the WHO framework. Time use in the healthy elderly is understood and should be considered when increasing the active ageing level of future elderly generations. More time spent on leisure and social participation would improve satisfaction and quality of life for elderly people in the moderate and low active ageing levels.

## Figures and Tables

**Table 1:** Characteristics of the elderly (*N* = 140).

Characteristics	*N*	(%)
Age (years)		
60-69	84	60.00
70-79	45	32.14
>80	11	7.86
Gender		
Male	29	20.71
Female	111	79.29
Marital status		
Single	8	5.71
Married	69	49.29
Widowed	63	45.30
Education		
Uneducated	10	7.14
Less than high school	111	79.29
High school graduate	9	6.43
Some college	7	5.00
College graduate (bachelor's degree)	3	2.14
Religion		
Buddhism	140	100.00
Chronic health conditions		
No	35	25.00
Yes^∗^	105	75.00
Current working status		
Not working	81	57.85
Working	59	42.15
Family status		
Single parent family	76	54.29
Extended family	64	45.71

^∗^Chronic health conditions include any of the following: heart disease, hypertension, arthritis, or diabetes.

**Table 2:** Number and percentage of elderly persons in the Community Elderly School, classified by active ageing index levels (*N* = 140).

Active ageing levels	*N*	%	AAI index		
			Min	Max	Mean ± SD
High	82	58.57	0.80	1	0.87 ± 0.05
Moderate	55	39.29	0.52	0.79	0.69 ± 0.08
Low	3	2.14	0.38	0.49	0.45 ± 0.06
Total	140	100.00	0.38	1	0.79 ± 0.06

**Table 3:** Number and percentage of elderly persons in the Community Elderly School, classified by active ageing index levels and characteristics (*N* = 140).

Characteristics	Active ageing level						Chi-square
	High		Moderate		Low		
	*N*	%	*N*	%	*N*	%	
Age (years)							0.09
60-69	47	33.57	35	25.00	2	1.43	
70-79	27	19.2	17	12.14	1	0.71	
>80	8	5.71	3	2.14	0	0	
Gender							0.04^∗^
Male	18	12.86	10	7.14	1	0.71	
Female	64	45.71	45	32.14	2	1.43	
Marital status							
Single	4	2.862	4	2.86	0	0	0.22
Married	38	7.14	29	20.71	2	1.43	
Widowed	40	28.57	22	15.71	1	0.71	
Education							
Uneducated	5	3.57	5	3.57	0	0	0.10
Less than high school	67	47.86	42	30.00	2	1.43	
High school graduate	5	3.57	4	2.86	0	0	
Some college	3	2.14	3	2.14	1	0.71	
College graduate (bachelor's degree)	2	1.43	1	0.71	0	0	
Religion							—
Buddhism	82	58.57	55	39.29	3	2.14	
Chronic health conditions							0.08
No	23	16.43	12	8.57	0	0	
Yes^∗^	59	42.14	43	30.71	3	2.14	
Current working status							0.22
Not working	48	34.29	33	23.57	0	0	
Working	34	24.28	22	15.71	3	2.14	
Family status							0.10
Single parent family	41	29.29	34	24.29	1	0.71	
Extended family	41	29.29	21	15.00	2	1.43	

^∗^ *p* < 0.05.

**Table 4:** Time use of daily occupations (*N* = 140).

Activities	*N*	%	Min (24 hours)	Max (24 hours)	Mean (24 hours)	Standard deviation
Basic ADL	140	100.00	1.13	6.67	2.34	0.75
Instrumental ADL	138	98.57	0.25	9.50	2.79	1.69
Rest and sleep	140	100.00	3.50	13.25	8.58	1.79
Education	3	2.14	1.75	2.25	2.00	0.25
Work	80	57.14	0.25	12.00	4.89	3.17
Leisure	138	98.57	0.25	13.50	6.34	3.17
Social participation	65	46.43	0.25	7.17	2.67	1.63

**Table 5:** Comparative time use with other ageing studies.

Activities	Thailand This study	France [22]	Canada [19]	USA [23]
	Mean (24 hours)			
Basic ADL	2.34	(Data unavailable)	2.26	2.71
Instrumental ADL	2.79	2.40	3.56	2.62
Rest and sleep	8.58	(Data unavailable)	7.89	8.80
Education	2.00	1.50	0.54	(Data unavailable)
Work	4.89	7.40	4.57	0.87
Leisure	6.34	4.10	3.41	6.17
Social participation	2.67	2.70	1.74	1.88

## Data Availability

The data availability and its supporting information files are contained within this article.
